# High Doses of D-Chiro-Inositol Alone Induce a PCO-Like Syndrome and Other Alterations in Mouse Ovaries

**DOI:** 10.3390/ijms22115691

**Published:** 2021-05-26

**Authors:** Arturo Bevilacqua, Jessica Dragotto, Micaela Lucarelli, Giovanna Di Emidio, Giovanni Monastra, Carla Tatone

**Affiliations:** 1 Department of Dynamic, Clinical Psychology and Health, Sapienza University of Rome, 00185 Rome, Italy; 2 Systems Biology Group Lab., The Experts Group on Inositols in Basic and Clinical Research (EGOI), 00185 Rome, Italy; g.monastra@gmail.com; 3 Department of Neuroscience, European Brain Research Institute (EBRI), Rome 00185, Italy; jessica.dragotto@gmail.com (J.D.); micaela.lucarelli@uniroma1.it (M.L.); 4 Department of Life, Health and Environmental Sciences, University of L’Aquila, 67100 L’Aquila, Italy; giovanna.diemidio@univaq.it (G.D.E.); carla.tatone@univaq.it (C.T.)

**Keywords:** inositol, letrozole, mouse ovary, PCOS model, aromatase, testosterone, androgenic phenotype, menopause

## Abstract

Administration of 1000–1500 mg/day D-Chiro-Inositol (DCIns) or a combination of Myo-Inositol (MyoIns) and DCIns in their plasma molar ratio (40:1) for three or more months are among recommended treatments for metabolic syndrome and/or Polycystic Ovary Syndrome (PCOS). We previously confirmed the efficacy of this formulation (8.2 mg/day MyoIns and 0.2 mg/day DCIns for 10 days) in a mouse PCOS model, but also observed negative effects on ovarian histology and function of formulations containing 0.4–1.6 mg/day DCIns. We therefore analyzed effects of higher doses of DCIns, 5, 10 and 20 mg/day, administered to young adult female mice for 21 days, on ovarian histology, serum testosterone levels and expression of the ovarian enzyme aromatase. Five mg/day DCIns (human correspondence: 1200 mg/day) altered ovarian histology, increased serum testosterone levels and reduced the amount of aromatase of negative controls, suggesting the induction of an androgenic PCOS model. In contrast, 10–20 mg/day DCIns (human correspondence: 2400–4800 mg/day) produced ovarian lesions resembling those typical of aged mice, and reduced serum testosterone levels without affecting aromatase amounts, suggesting a failure in steroidogenic gonadal activity. Notwithstanding physiological/biochemical differences between mice and humans, the observed pictures of toxicity for ovarian histology and function recommend caution when administering DCIns to PCOS patients at high doses and/or for periods spanning several ovulatory cycles.

## 1. Introduction

Polycystic Ovary Syndrome (PCOS), the most common endocrine disorder in women of reproductive age, is diagnosed by the presence of two of the following disorders: hyperandrogenism, oligo/anovulation and polycystic ovaries, with the exclusion of other related pathologies, according to the 2003 Rotterdam Criteria [1].

The different features and pathophysiology of PCOS, which include genetic [2] and environmental/organic factors, such as endocrine disruptors [3], obesity and an imbalance in diet composition [4], have long been approached in experimentally modeled mammals [5,6]. Rodent models of PCOS are produced using various procedures [7,8]. For example, a hyperandrogenism-dependent PCOS-like syndrome, with a failure in folliculogenesis, may be obtained by administration of androgens [9,10,11] or of the aromatase inhibitor letrozole [12], which prevents the physiological conversion of androgens to estradiol inside the ovarian follicle. Letrozole-conditioned mice are infertile and recapitulate most of reproductive and metabolic aspects of PCOS [13]. Alternatively, rodents can be modeled by exposure to a regimen of continuous light, which disrupts the normal light–dark cycle and the circadian rhythm of melatonin, lowering peak levels of gonadotropins and progesterone [14,15], and producing, among others, oligo-anovulation, polycystic ovaries with hyperplastic follicular theca-cell layers [16], follicular atresia [17] and infertility [16].

Insulin resistance plays an important role in the pathophysiology of PCOS [18] and a severe deregulation of inositol metabolism in follicle cells of PCOS patients is largely acknowledged [19]. The two main inositol isomers, myo-inositol (MyoIns) and D-chiro-inositol (DCIns), are precursors of inositol phosphoglycans (MyoIns-IPG and DCIns-IPG) that act as second messengers of insulin [20,21,22]. A growing body of research has shown that MyoIns and DCIns can be synergistically integrated in the clinical management of PCOS, exerting therapeutic effects and representing a reliable alternative to conventional treatments for insulin resistance [23,24,25,26], if combined in amounts corresponding to their physiological plasma molar ratio of 40:1 [27,28] at doses of approximately 2g twice a day [27].

Our group has recently confirmed the efficacy of the 40:1 MyoIns/DCIns formulation in the continuous light-induced PCOS mouse model. In fact, a ten-day treatment with 410 mg/kg/day MyoIns and 10 mg/kg/day DCIns, corresponding to 8.4 mg total inositol/mouse/day, restored the normal ovarian histology and fertility [16]. In contrast, formulations with MyoIns/DCIns ratios of 20:1 and 5:1, with DCIns amounts of, respectively, 20 and 70 mg/kg/day, worsened the PCOS-like ovarian features and extended over time the infertility status of the mice. This agrees with the observations that the ovarian MyoIns/DCIns molar ratio is maintained around the value of 100:1 in healthy women but drops to 0.2:1 in PCOS women [29] and that high levels of DCIns in the follicular fluid of patients enrolled in an IVF program are harmful for oocyte and blastocyst quality [30,31].

With these premises and in light of the observation that DCIns reduces aromatase expression in human granulosa cells [32], we hypothesized that, similarly to the effects of the aromatase inhibitor letrozole, the administration of DCIns alone at high doses to normal female mice would result in the production of an androgenic PCOS-like model or other ovarian lesions.

We tested this hypothesis by administering DCIns to wild-type female mice for three weeks, approximately corresponding to five ovulatory cycles, and analyzing the effects on ovarian histology/function, serum testosterone levels and expression of the ovarian enzyme aromatase. To this end, we employed three different DCIns formulations: 250 mg/kg/day, 500 mg/kg/day and 1000 mg/kg/day. These doses provide approximate daily amounts of 5 mg, 10 mg and 20 mg DCIns/mouse, which are comparable to human doses of 1200, 2400 and 4800 mg/day, respectively [33].

The lowest dose is in the range of 1000 to 1500 mg used in some therapeutic regimens of PCOS patients [34,35]. Higher doses were employed to construct a dose response-curve of possible DCIns toxicity.

The present paper describes histological and hormonal features observed in this study, having particular relevance under a gynecological point of view. Effects of DCIns at a cellular and molecular level are still being investigated in our laboratories and will be reported elsewhere. On the one hand, mice that received 250 mg/kg/day DCIns or 500 μg/kg/day letrozole (positive controls) modeled a PCOS-like syndrome, with cystic ovaries, high testosterone levels and low amounts of ovarian aromatase; on the other hand, mice that received 500 mg/kg/day and 1000 mg/kg/day DCIns displayed histological ovarian lesions resembling a menopausal state, with increased follicular/stromal cellularization and loss of normal follicular structure, low testosterone levels and unaltered amounts of ovarian aromatase.

## 2. Results

### 2.1. Increase in Mouse Weights during the Treatment

**Figure 1:**
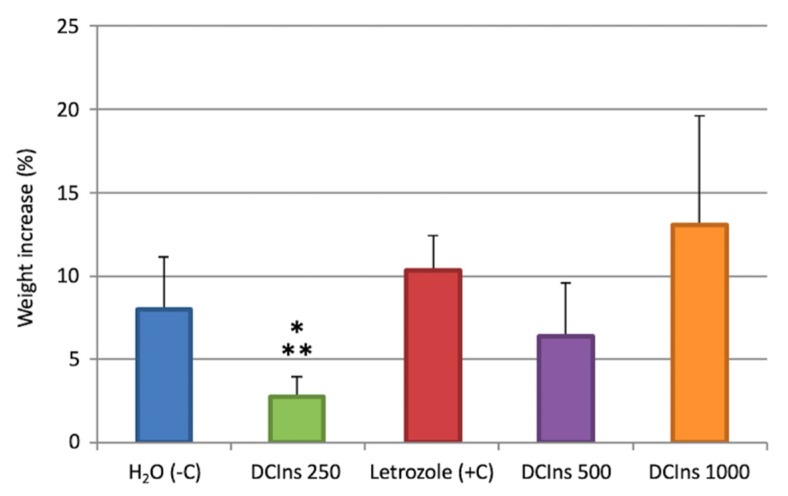
Increase in mouse weights during the 21-day treatment. Histograms represent percent weight increases (mean ± SD) depending on the molecules and doses received. One Way ANOVA, *p* < 0.005; *, *p* < 0.05 versus H_2_O and letrozole; **, *p* < 0.01 versus DCIns 1000.

During the 21-day treatment, the weights of DCIns 250-mice increased 2.73 ± 1.22%, from 23.15 ± 2.8 g to 23.78 ± 2.96 g (mean ± SD), which was less than negative control mice (8.0 ± 3.16%, from 21.02 ± 2.77 g to 22.70 ± 2.69 g) and letrozole-treated positive control mice (10.33 ± 2.13%, from 19.48 ± 2.56 g to 21.46 ± 2.60 g). The weights of DCIns 500-treated mice and DCIns 1000-treated mice increased respectively by 6.40 ± 3.18%, from 19.64 ± 2.60 g to 20.86 ± 2.43 g, and 13.06 ± 6.53%, from 20.78 ± 2.98 g to 23.39 ± 2.54 g (Figure 1). In the post-hoc Tukey HSD test, the weight increase of DCIns 250-treated mice was different from those of negative controls or letrozole-treated mice (*p* < 0.05) and DCIns 1000-treated mice (*p* < 0.01).

### 2.2. Assessment of Cycle Progression during the Treatment

Daily evaluations of the estrus cycle revealed a progression through all stages in the negative control group; none of the mice arrested (0/5). On the contrary, the cycle was arrested at day 8–10 during other treatments in 3/5 mice (DCIns 250), 3/5 mice (DCIns 500), 4/5 mice (DCIns 1000) and 4/5 mice (positive controls).

### 2.3. Gross Morphology of Uteri/Ovaries and Histology of Ovaries at the End of the Treatment

**Figure 2:**
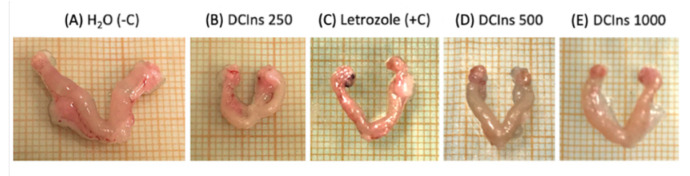
Macroscopic view of mouse uterus-ovary complexes at the end of the 21 day-treatment. Gross morphology of typical uteri and ovaries from mice that received, from left to right: (**A**) plain water (negative control), (**B**) DCIns 250, (**C**) letrozole (positive control), (**D**) DCIns 500 and (**E**) DCIns 1000. Note the longer extension and thicker appearance of a typical uterus from control mice (**A**) compared with the shorter and thinner appearance of the uteri from DCIns- and letrozole-administered mice (**B**–**E**). All uteri are shown at the same scale (mm).

Inspected visually after the three week-treatment (Figure 2), uteri of control mice (Figure 2A) displayed a proestrus/estrus-like aspect, typical of mature and cycling animals.

Uteri of mice that had received either DCIns (Figure 2B,D,E) or letrozole (Figure 2C) had an immature/metestrus-diestrus-like aspect, typical of non-cycling animals. Ovaries from control mice displayed the presence of large follicles and some corpora albicantia, products of recent ovulation. Ovaries of both DCIns- and letrozole-treated mice had a smaller and immature size, but occasional presence of corpora albicantia was observed.

**Figure 3:**
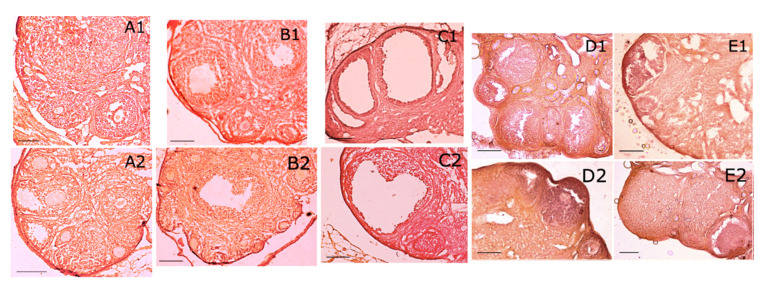
Ovarian histology of mice at the end of the 21 day-treatment. (**A1**,**A2**) Ovarian sections from mice that received plain water (negative control), showing primary, secondary, and tertiary follicles as well as a corpus luteum (**A1**). These features are typical of normally cycling mice. (**B1**,**B2**) Sections from mice subjected to 21-day treatment with DCIns 250, showing primary, secondary, tertiary and cystic follicles devoid of oocytes. These features resemble typical signs of PCOS. (**C1**,**C2**) Sections from mice subjected to 21-day treatment with letrozole (positive controls), showing paucity of follicles and a large cyst, typically modeling human PCOS. (**D1**,**D2**) Sections from mice subjected to 21-day treatment with DCIns 500, showing secondary and tertiary follicles as well as follicles with signs of hyperproliferation and extension of the stromal compartment. (**E1**,**E2**) Sections from mice subjected to 21-day treatment with DCIns 1000, showing paucity of secondary and tertiary follicles as well as large follicles with signs of hyperproliferation and extension of the stromal compartment. Hematoxylin-eosin. Scale bars, 100 μm.

**Figure 4:**
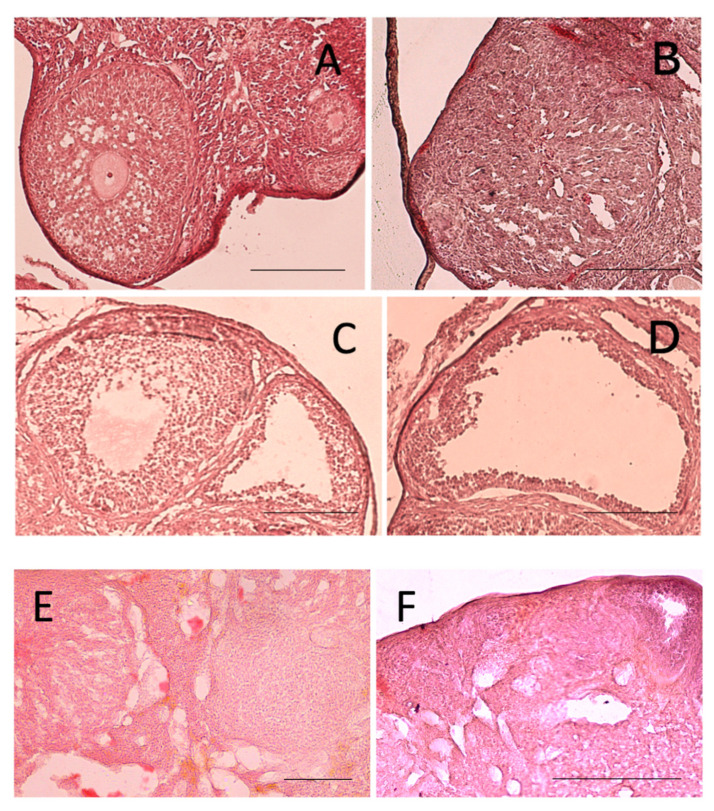
Histological features of mouse ovaries at the end of the 21 day-treatment. (**A**,**B**) Ovarian sections from mice that received plain water (negative control), showing a primary and a tertiary follicle (**A**) and a corpus luteum (**B**). (**C**) A section from a DCIns 250-treated mouse with cysts. (**D**) A section from a letrozole-treated mouse (positive control) with large cysts. (**E**) A section from a DCIns 500-treated mouse with follicular hyperproliferation. (**F**) A section from a DCIns 1000-treated mouse with stromal extension. Hematoxylin-eosin. Scale bars, 100 μm.

Histology of ovaries from negative control mice showed a normal presence of primary, secondary, tertiary follicles containing a growing oocyte, and of corpora lutea (Figure 3A1,A2; Figure 4A,B).

Ovaries from DCIns 250-treated mice had apparently normal primary and secondary follicles but also cystic tertiary follicles, some of which contained an atretic oocyte, strongly resembling those found in human polycystic ovaries (Figure 3B1,B2; Figure 4C). Ovaries of letrozole-treated mice were similar but contained larger cystic follicles characterized by the absence of the oocyte (Figure 3C1,C2; Figure 4D). In some cases, secondary and tertiary follicles from these ovaries had a clearly atretic oocyte with cytosolic vacuolization, suggesting the progression of an apoptotic process.

Ovaries from DCIns 500- and DCIns 1000-treated mice had some primary and secondary follicles, a very limited number of tertiary follicles, no follicles at more advanced stages and no cystic follicles (Figure 3D1,D2; Figure 3E1,E2). These ovaries had large follicles/areas with diffused, aberrant cell proliferation (Figure 4E). The typical structure of the ovary was lost, especially under the DCIns 1000 dose, and the presence of follicles was difficult to assess in many cases (Figure 4F). In some sections, the presence of oedema foci was observed.

### 2.4. Follicular Composition of Mouse Ovaries at the End of the Treatment

**Figure 5:**
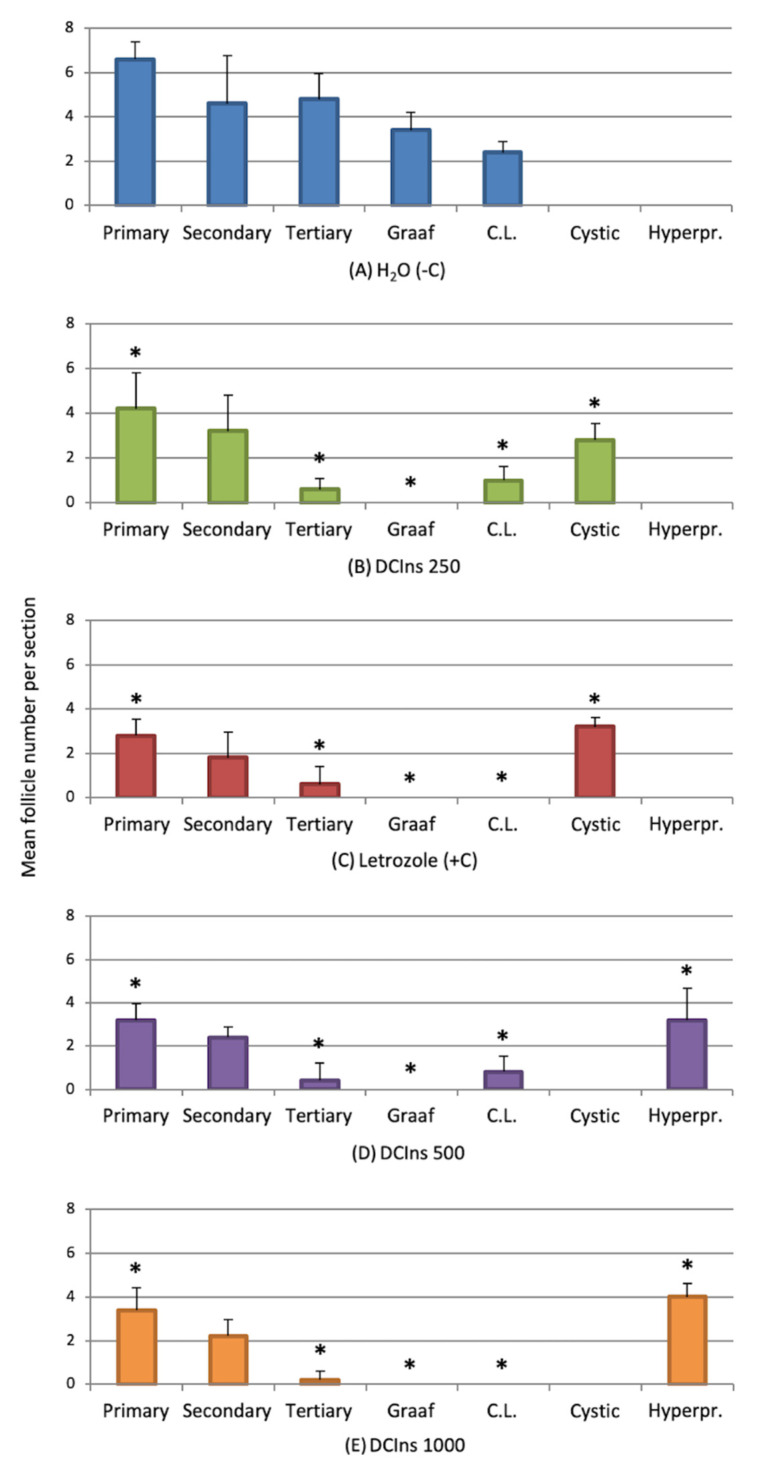
Number of ovarian follicles at different stages and of different types at the end of the treatment. Histograms represent numbers of follicle of various types in abscissa (mean ± SD). *, difference in group composition vs. negative control ovaries; *p* < 0.05, calculated by repeated measures ANOVA.

Complete ovarian sections from all mice were used to calculate the number of developing follicles at each stage (Figure 5).

Negative control ovaries (A) displayed comparable numbers of primary, secondary and tertiary follicles, and graafian follicles and corpora lutea were also present, although in a smaller amount. Ovaries from DCIns 250- (B) and letrozole-treated mice (C) displayed progressively decreasing numbers of growing follicles and presence of cystic follicles. Corpora lutea were present in DCIns 250-ovaries but not letrozole-ovaries. At the DCIns 500 (D) and 1000 doses (E), ovaries displayed a similar decrease in small follicles, no cystic follicles but larger follicles with evidence of cellular hyperproliferation and areas of cellular necrosis.

### 2.5. Theca/granulosa Cell Layer Measurements and Their Ratio

**Figure 6:**
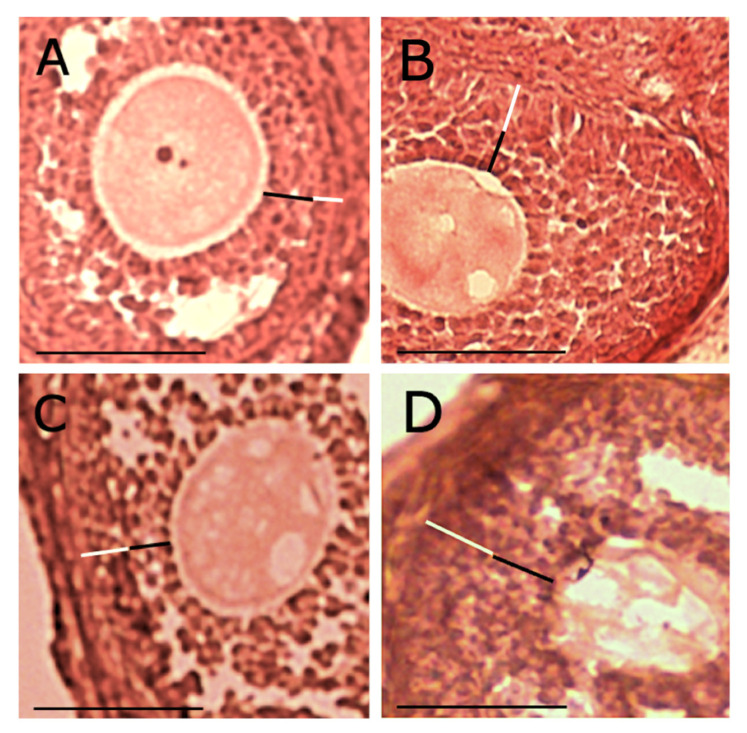
Extension of theca and granulosa cell layers in ovarian follicles. (**A**) negative control mice; (**B**) mice subjected to 21-day treatment with DCIns 250; (**C**) mice subjected to 21-day treatment with letrozole; and (**D**) mice subjected to 21-day treatment with DCIns 500. The thickness of granulosa cell- (black bars) and theca cell-layers (white bars) is marked. Hematoxylin-eosin. Scale bars, 50 μm. Note the different aspect of oocytes in all panels.

The DCIns 250 dose induced cystic follicles containing a hyperplastic layer of theca cells and variable amounts of granulosa cells. Cystic follicles of letrozole-treated mouse ovaries also had a thin layer of somatic cells. Early tertiary follicles from ovaries of mice under all experimental conditions were assayed for the extension/thickness of the theca and granulosa cell layers (Figure 6) and to calculate their ratio (TGR) (Table 1).

As shown, the granulosa cell compartment of follicles typical of control mouse ovaries had a thicker extension than the theca cell compartment, and the relative TGR value (0.48 ± 0.05) was in the range of normality observed previously [16]. In contrast, the theca cell compartment of ovarian follicles from DCIns 250-, DCIns 500- and letrozole-treated mice was thicker than the granulosa cell one with TGR values of, respectively, 1.02 ± 0.13, 1.26 ± 0.09 and 1.13 ± 0.15. Interestingly, the TGR of DCIns 250-treated mice was different from that of DCIns 500-treated ones, suggesting a dose-dependent worsening effect of DCIns. It was impossible to calculate a TGR of DCIns 1000-treated mice due the extreme paucity of regular early tertiary follicles in their ovaries.

### 2.6. Testosterone Levels at the End of the Treatment

Under the DCIns 250 dose, levels of testosterone (1.42 ± 0.107 ng/mL) (mean ± SD) increased above those typical of negative control mice (0.37 ± 0.11 ng/mL) (*p* < 0.05, one-way ANOVA), similarly to those of letrozole-administered mice (1.29 ± 0.22 ng/mL). Levels of testosterone in the serum of DCIns 500- and DCIns 1000-treated mice were similar to or lower than those of negative control mice, being respectively 0.14 ± 011 and 0.27 ± 0.37 ng/mL.

### 2.7. Presence and Relative Amounts of Aromatase at the End of the Treatment

**Figure 7:**
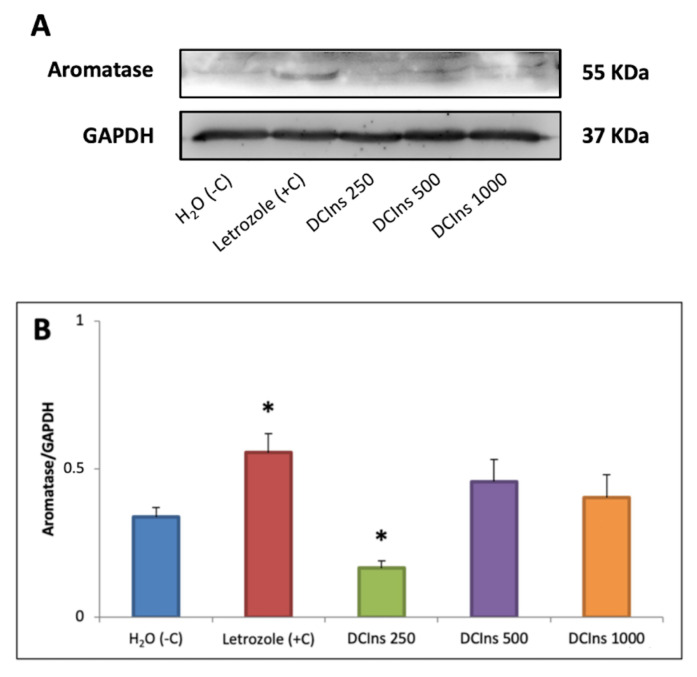
Levels of aromatase in the ovaries of mice at the end of the 21-day treatment. (**A**) Representative Western blot of aromatase in protein extracts from the ovaries of mice under the experimental conditions indicated. (**B**) Densitometric analysis of aromatase/GAPDH. Values represent the mean value ± SD of extracts from three independent experiments. *, difference vs. negative control mice, *p* < 0.05.

Presence of aromatase in the ovaries of treated and negative control mice was evaluated by Western blot analysis and subsequent densitometry, normalized by comparison with ubiquitous GAPDH (Figure 7).

From a qualitative point of view, a single band of approximately 55 kDa molecular mass was detected in protein extracts from control mouse ovaries, as already reported [36]. A band of similar size was detected after all treatments, but additional bands were detected, as follows: protein extracts from DCIns 250-treated mice contained a band of smaller size (approximately 52 kDa), those from letrozole-treated mice had a band of closely smaller size (approximately 54 kDa), and extracts from DCIns 500- and DCIns 1000-treated mice had a band of much smaller size (approximately 50 kDa). Induction of different aromatase isoforms by various treatments in mouse ovarian cells appears reasonable in light of the observation that the control of aromatase expression in humans involves a complex mechanism of different promoter and downstream exons [37]. Densitometric measurements of the amounts of aromatase bands relative to GAPDH, showed a reduction in DCIns 250-ovaries (0.16 ± 0.02 relative units, RU) (mean ± SD) from those of negative control-ovaries (0.34 ± 0.03 RU) (*p* < 0.05); on the contrary, an increase was observed in letrozole-ovaries (0.56 ± 0.06 RU) (*p* < 0.05). No variations in the amounts of aromatase were observed in DCIns 500-ovaries (0.45 ± 0.07 RU) and DCIns 1000-ovaries (0.40 ± 0.08 RU) from either negative control- or letrozole-ovaries.

## 3. Discussion

### 3.1. Effects of the Administration of DCIns 250 and Letrozole

Under the 5 mg/day DCIns dose employed, mice developed distinct morphological features of human PCOS, similar to those observed in mice that received 10 μg/day letrozole, an inducer of androgenic PCOS models [12,38]. Their uterus had the macroscopic aspect typical of non-cycling animals, in agreement with the cycle arrest observed in 3/5 mice and suggestive of an impairment of gonadal steroidogenic activity.

Compared with negative control mouse ovaries, those of DCIns 250-treated mice and of letrozole-treated mice had progressively decreasing numbers of follicles at the primary, secondary, tertiary stages and corpora lutea, absence of Graafian follicles and presence of cystic follicles. The TGR of follicles of DCIns 250- and letrozole-treated mice was also similar to that typical of PCOS mouse follicles [16], confirming an extension of the theca cell compartment and a reduction of the granulosa cell one. The androgenic phenotype observed with these treatments was confirmed by measurements of blood testosterone levels of the mice, which were increased several-fold with respect to those of negative control mice. Either an overproduction of androgens in the extended theca cell compartments and/or a specific decrease in aromatase activity is probably responsible for this increase. Additionally, it may depend on the reduced granulosa cell compartment measured under these conditions. The observed increase in blood testosterone was associated with a decrease in expression of aromatase in the ovary of DCIns 250-treated mice. This finding represents the first evidence of a specific down modulation of aromatase by DCIns in an in vivo system and agrees with similar observations on in vitro cultured human granulosa cells [32]. In contrast, a similar rise in blood testosterone observed in letrozole-treated mice was associated with increased ovarian aromatase expression. This apparently paradoxical observation is in accordance with the existence of a letrozole-induced positive regulatory feedback [13] that involves high levels of circulating testosterone and elevated expression of FSH receptors and aromatase.

In summary, 250 mg/kg/day DCIns for 21 days gave a murine model with major ovarian and hormonal features of human PCOS.

Finally, DCIns 250-treated mice displayed a significantly decrease in body weight gain with respect to other conditioned mice, including positive and negative controls, as observed in other PCOS mouse models [16,39]. The reduced weight gain of these mice could not be attributed to the lipolytic effects of increased androgen levels, considering the increased weight gain observed in letrozole-treated mice, but suggests the existence of a specific metabolic effect(s) of the DCIns 250 dose, possibly acting by reduction of food intake, as shown by Jeon and coworkers [40]. Whether this is due to an abnormal interaction with the synthesis of appetite-signaling peptides deserves further investigation.

These results may be interpreted by considering the specific biochemical actions of inositols in the ovary. Body fluids, organs and tissues maintain specific molar ratios in the content of MyoIns and DCIns [41], mostly by the tissue-specific, insulin-dependent activity of the enzyme epimerase. This enzyme irreversibly converts MyoIns into DCIns, according to the functions and specific metabolic requirements of the cell types. Since MyoIns increases cellular glucose uptake, while DCIns is crucial for glycogen synthesis [42], glycogen-storage organs/tissues, such as muscle, fat, and liver, require high levels of DCIns; in contrast, organs displaying high glycolytic activity, such as brain, heart, and ovary, contain very low levels of DCIns [19,43,44,45]. PCOS ovaries, having an inverted MyoIns/DCIns ratio due to epimerase hyperactivation [45], confirm the ovarian requirement of high amounts of MyoIns and low amounts of DCIns. In fact, MyoIns concentration in the mammalian female reproductive tracts is substantially higher than in plasma [46] and a deficiency of MyoIns is known to impair oocyte and embryo quality [47]. On the contrary, supplementation of MyoIns to in vitro cultured mouse oocytes and embryos enhances their ability to complete preimplantation development [47,48] and to develop to term normally [49]. MyoIns (such as inositol trisphosphate, IP_3_) is one of the second messengers of FSH in the ovary [50], relevant for granulosa cell function and folliculogenesis. For where oocytes are concerned, MyoIns-derived IP_3_ modulates intracellular Ca^2+^ effluxes in response to LH and FSH [51,52] and plays a key role in meiotic maturation [53]. Therefore, as predicted several years ago [54], high doses of MyoIns can be administered to PCOS patients with positive effects on ovarian and oocyte/embryo functions.

High doses of DCIns, on the contrary, affect ovarian functions negatively and worsen PCOS symptoms. As a matter of fact, the only known roles of DCIns are to participate in insulin signal transduction downstream of the insulin receptor and, in the ovary, to stimulate testosterone synthesis initiating steroidogenesis in theca cells [55] and, as shown here, to downregulate aromatase, reducing the conversion of androgens into estrogens. Therefore, while at the systemic level, high doses of DCIns improve insulin activity, reducing its levels and counteracting insulin resistance, at the ovarian level they cause a switch to an androgenic phenotype and the consequent impairment in folliculogenesis and overall function [19,30]. The existence of additional abnormal pathways induced by an excess of ovarian DCIns warrants further investigation.

These results deserve particular attention since, although being in apparent contrast with a positive outcome of the therapy for human PCOS with a comparable dose of DCIns (1200 mg/day), they support the general conclusion of clinical trials that, although describing positive results only on metabolic parameters, doubt remains on the effects on ovarian function [34,55].

### 3.2. Effects of the Administration of DCIns 500 and DCIns 1000

Under the DCIns doses of 10 mg/day and 20 mg/day, corresponding to 2400 mg/day and 4800 mg/day in humans, mice had macroscopic uteri typical of non-cycling mice, and a cycle arrest detected in 3/5 and 4/5 mice, respectively, suggesting an impairment in gonadal steroidogenic activity by these treatments as well.

Ovarian histology of these mice, however, showed substantial differences. In particular, large areas of ovarian sections were occupied by diffuse cell populations, probably induced by active and aberrant proliferation, in both the stroma and abnormally expanded follicles. In several cases, these follicles, here named “hyperproliferative”, displayed signs of necrosis, suggesting the failure of metabolic processes.

Ovaries of DCIns 500- and DCIns 1000-treated mice resembled those from aged mice [56,57], or mice experimentally induced to a menopausal state [58], with paucity or absence of follicles and presence of signs of general cell proliferation. Some of their features also resemble ovarian abnormalities in a pathological model of ovarian lesions induced in rats by administration of raloxifene, a selective estrogen receptor modulator, for 6 months [59]. These include, among others, a dramatic decrease in the number of developing follicles and corpora lutea, presence of anovulatory follicles with atretic oocytes, lack of corpora lutea, hyperplasia of granulosa cells, ovarian atrophy and failure in ovulation.

The disruption of the ovarian organization was associated with minimal levels of testosterone measured in the serum of DCIns 500- and DCIns1000-treated mice, similar to or lower than negative control mice. Since the ovarian content of aromatase under both experimental conditions was similar to that of negative control mice, we hypothesize that these treatments block normal hormonal pathways inside the ovary, probably by inhibiting expression/activity of the steroidogenic enzyme cytochrome P450scc that catalyzes the initial step in steroidogenesis.

## 4. Materials and Methods

### 4.1. Animals

Twenty-five 30-day-old inbred C57BL/6N female mice (Charles River Italia, Calco, VA, Italy), housed in a temperature-controlled facility (22 ± 1 °C) on a 12/12 h light/dark cycle, inside standard cages with unlimited access to food and water, were used for all experimental treatments. All efforts were made to minimize animal suffering, according to the European directive 2010/63/EU and the Italian law DL 26/2014 on the protection of animals used for scientific purposes. Protocols were approved by the University of L’Aquila Organism for Animal Welfare (OPBA) and by the Italian Ministry of Public Health with authorization n. 269/2018-PR (April 9^th^, 2018) to Carla Tatone. 

### 4.2. Administration of DCIns or Letrozole

To evaluate the effects of DCIns or letrozole, mice were randomly divided into five cages with five animals per cage. Each cage was provided with a bottle of water containing 5 mg/2 mL or 10 mg/2 mL or 20 mg/2 mL DCIns, 2 mL being the average volume of water drunk by a single 20 g mouse per day, previously recorded [16]. Positive control mice received 10 μg/2 mL letrozole (Sandoz Italia, Origgio, Italy), corresponding to 0.5 mg/kg [12]. Negative control mice received plain drinking water. All mice were kept under free feeding and drinking conditions for 21 days with bottle replacements every two to three days and weighed weekly during the treatment.

### 4.3. Vaginal Smears

Starting from the second week of treatment, all mice were subjected to daily evaluation of the progression of their estrus cycles by a direct, “wet smear” technique [10,60]. Vaginal cells were collected via saline lavage with a plastic pipette filled with 10 μL phosphate-buffered saline (PBS), and observed without staining under a light transmission microscope (Leica DMLB, Leica Microsystems GmbH, Wetzlar, Germany), with a 10× objective. Predominant nucleated epithelial cells and some cornified epithelial cells indicated the proestrus stage; predominant cornified squamous epithelial cells indicated the estrus stage; cornified squamous epithelial cells and leukocytes indicated the metestrus stage; predominant leukocytes indicated the diestrus stage.

### 4.4. Blood and Organ Collection

At the end of the treatment, all mice were sacrificed by an inhalant overdose of carbon dioxide (CO_2_, 10–30%), followed by cervical dislocation for histological and biochemical analyses.

For each mouse, the blood was rapidly drawn from the heart and prepared for testosterone assay; the uterus and ovaries were excised and photographed; one ovary was processed for histological analysis, the other ovary was frozen in a mixture of dry ice/70% ethanol for biochemical analyses.

### 4.5. Histological Analysis

Ovarian histology was performed as described [16]. Organs were thawed, fixed in 4% paraformaldehyde at 4 °C overnight, washed in PBS, dehydrated in 30% sucrose and embedded in paraffin. Five or 10 μm thick sections were mounted on gelatinized slides, stained with hematoxylin-eosin (H&E), cover-slipped with Eukitt^®^ and observed under the Leica DMLB microscope. The number of primary, secondary and tertiary follicles in complete ovarian sections from three animals per treatment were recorded for statistical analysis. The thickness of theca cell and of granulosa cell layers of early tertiary follicles from various sections was measured using the ImageJ software (ImageJ 1.47v, Wayne Rasband, National Institutes of Health; Washington, DC, USA).

### 4.6. Serum Testosterone Assay

Serum from each mouse was obtained by allowing 100 μL blood to clot for 1 h at room temperature (RT) and then centrifuging the sample for 10 min at 1500 g at 4 °C. Testosterone serum concentrations were measured using a mouse ELISA assay (Intra-Assay: CV < 8%, Inter-Assay: CV < 10%; FineTest, Wuhan, China).

### 4.7. Western Blot Analysis of Ovarian Aromatase

For protein extraction, one ovary from each mouse, freed of adipose tissue and blood, was homogenized in RIPA lysis buffer [61] containing protease and phosphatase inhibitors (Sigma–Aldrich, Milano, Italia), by repeated freezing/thawing cycles in liquid nitrogen and centrifuged at 10,000× *g* for 30 min a 4 °C. Soluble protein concentration in the supernatant was determined by BCA protein assay kit (Pierce, Rockford, IL, USA). Twenty micrograms of protein from each sample was separated by SDS-PAGE and transferred to a polyvinylidene difluoride membrane (Sigma-Aldrich, St. Louis, MO, USA). Non-specific binding sites were blocked for 1 h at RT with 5% BSA in Tris-buffered saline containing 0.05% Tween 20 (TBS-T). Identification of aromatase, and glyceraldehyde 3-phosphate dehydrogenase (GAPDH) as the internal standard, was performed by membrane incubation with polyclonal rabbit anti-aromatase (PA1-21398, Thermo Fisher Scientific, Waltham, MA, USA; 1:500) and mouse anti-GAPDH (TA802519, OriGene Technologies Inc., 1:750) antibodies, overnight at 4 °C, followed by incubation with horseradish peroxidase (HRP) conjugated anti-rabbit (7074S, Cell Signaling Technologies, Danvers, MA, USA) 1:5000) or anti-mouse secondary antibody (Ab6728, Abcam, 1:5000), respectively, for 1 h at RT. After washing in TBS-T, specific immunoreactive complexes were detected by ECL kit (Thermo Fisher Scientific) and Uvitec Cambridge system (Alliance series, Cambridge, UK). Bands were normalized for GAPDH using ImageJ 1.47v software. Experiments were performed in triplicate.

### 4.8. Chemicals

Where not stated otherwise, chemicals were purchased from Sigma-Aldrich Co. (St. Louis, MO, USA). DCIns (96.53%) was provided by Amicogen Inc. (Jinju-si, Korea).

### 4.9. Statistical Analysis

Data were analyzed by one-way or repeated measures analysis of variance (ANOVA). Post-hoc analyses were performed by the Tukey Honestly Significant Differences test. Statistical analyses were performed using R: A language and environment for statistical computing (R development core team, R foundation for statistical computing, ISBN 3–900051-07-0, 2008, Vienna, Austria).

## 5. Conclusions

The overall results may be summarized as follows:

1. Exposure of mice to 5 mg/day DCIns for 21 days represents a novel procedure to obtain a useful experimental model of PCOS.
2. Exposure of mice to higher daily amounts of DCIns for 21 days is toxic for ovarian histology and function, producing lesions different from those typical of PCOS but resembling a pre-menopausal/menopausal state.
3. Serum testosterone levels are affected by administration of DCIns and letrozole. They are increased by 5 mg/day DCIns, but strongly decreased by higher DCIns amounts, probably due to a blockade in steroidogenesis produced by these doses.
4. The amounts of ovarian aromatase are affected by administration of both 5 mg/day DCIns and letrozole but in opposite direction: DCIns downregulates and letrozole upregulates aromatase expression, confirming previous observations. Higher DCIns doses do not affect the amount of ovarian aromatase.

Although recognizing species-specific differences between mice and humans, the present conclusions deserve attention for clinical practice. In fact, when administered for a prolonged time, human treatments with DCIns doses of 1200 mg/day or higher should be carefully evaluated for their possibly detrimental impact on ovarian physiology.

This study has several strengths related to the use of a quickly achievable animal model representative of the physio-pathological processes evaluated. We recognize one main weakness, consisting in the lack of data on serum levels of estrogens and other gonadal steroids, due to the paucity of blood that can be drawn by a single 20 to 30 g mouse for the type of hormonal assay employed.

Ongoing experiments in our laboratory are extending these observations, taking into consideration molecular and cellular aspects of ovarian abnormalities and a complete evaluation of hormonal parameters.

## Figures and Tables

**Table 1:** Thickness of theca (TC) and granulosa cell (GC) layers in control, DCIns- and letrozole-treated mice. Six representative early tertiary follicles from mice subjected to different experimental treatments were measured using the ImageJ software. The TC/GC Ratio (TGR) (mean, SD) is reported below.

H_2_0 (−C)		Letrozole (+C)		DCIns 250		DCIns 500	
TC	GC	TC	GC	TC	GC	TC	GC
0.101	0.23	0.106	0.109	0.1	0.121	0.115	0.083
0.116	0.22	0.1	0.081	0.11	0.093	0.096	0.078
0.097	0.21	0.098	0.088	0.097	0.103	0.105	0.088
0.11	0.255	0.09	0.075	0.131	0.12	0.11	0.081
0.12	0.26	0.095	0.071	0.124	0.129	0.114	0.093
0.135	0.25	0.102	0.108	0.107	0.097	0.1	0.086
TGR							
Mean	0.48		1.13 *		1.02 *		1.26 *^,^ **
SD	0.05		0.15		0.13		0.09

Note. Values were then converted in μm according to a micrometer eyepiece: 0.1 unit = 9 μm. One Way ANOVA, *p* < 0.001; * *p* < 0.01 versus H_2_O; ** *p* < 0.01 versus H_2_O; ** *p* < 0.05 versus DCIns 250.

## Data Availability

Data supporting present observations and results are available from the corresponding author upon reasonable request.
